# Tumor necrosis and complete resection has significant impacts on survival in patients with limited-stage upper aerodigestive tract NK/T cell lymphoma

**DOI:** 10.18632/oncotarget.18107

**Published:** 2017-05-23

**Authors:** Moo-Kon Song, Joo-Seop Chung, Ho-Young Yhim, Sung-Nam Lim, Seong-Jang Kim, Yeon-Hee Han, Hye-Kyung Shim, Sung-Hoon Jung, Je-Jung Lee, Deok-Hwan Yang

**Affiliations:** ^1^ Department of Hemato-Oncology, Hanyang University Hanmaeum Changwon Hospital, Changwon, Korea; ^2^ Department of Hematology-Oncology, Pusan National University Hospital, Busan, Korea; ^3^ Department of Hematology, Chonbuk National University Hospital, Jeonju, Korea; ^4^ Department of Hematology, Busan Haeundae Paik Hospital, Busan, Korea; ^5^ Department of Nuclear Medicine, Pusan National University Hospital, Busan, Korea; ^6^ Department of Nuclear Medicine, Chonbuk National University Hospital, Jeonju, Korea; ^7^ Department of Nuclear Medicine, Busan Haeundae Paik Hospital, Busan, Korea; ^8^ Department of Hematology-Oncology, Chonnam National University Hwasun Hospital, Hwasun, Korea

**Keywords:** extranodal natural killer/T-cell lymphoma, tumor necrosis, complete resection, prognosis

## Abstract

Tumor necrosis (TN) is associated with worse prognosis in several solid cancers. Whether TN predicts poor outcome in natural killer cell / T cell lymphoma (NKTCL) is unclear. We investigated the clinical impact of TN on survival and other novel prognostic parameters in upper aero-digestive tract (UAT) NKTCL of 100 patients with limited stage. TN was significantly associated with poor performance status (*p* = 0.049), high Korean Prognostic Index score (*p* = 0.024), high C-reactive protein/albumin ratio (*p* = 0.003), higher maximum standard uptake value on positron emission tomography/computed tomography (PET/CT) (*p* = 0.008) and higher metabolic tumor volume (MTV) on PET/CT (*p* < 0.001). In univariate and multivariate analyses, progression-free survival and overall survival were independently associated with High MTV status (*p* = 0.001, *p* = 0.032), TN (*p* = 0.018, *p* = 0.009), local tumor invasiveness (*p* = 0.007, *p* = 0.035), complete resection (*p* = 0.020, *p* = 0.028) and regional lymph node involvement (*p* < 0.001, *p* < 0.001). TN and complete resection are concluded to be novel independent prognostic factors in patients with UAT NKTCL.

## INTRODUCTION

Extranodal (EN) natural killer cell/T cell lymphoma (NKTCL) is a clinicopathologic disease entity with an aggressive clinical course [1, 2]. NKTCL usually involves the nasal cavity, nasopharynx and other upper aerodigestive tract (UAT) sites, although it can arise in any EN site [3-5].

Radiotherapy (RTx) is an important therapeutic modality in NKTCL, reflecting the radiosensitivity of the lymphoma. RT alone in early stage NKTCL can yield excellent overall and complete response rates [6]. However, systemic relapse after RTx alone occurs in up to 40 % of patients. Therefore, chemotherapy (CTx) combined with RTx has been used to improve clinical outcomes. Two recent prospective trials studied whether concurrent chemoradiotherapy can improve the clinical benefits of radio-sensitization in NKTCL [5, 7, 8]. The current guidelines of the National Comprehensive Cancer Network are equivocal regarding the optimal therapy for limited stage nasal NKTCL and include RTx alone, sequential CTx or concurrent chemoradiotherapy. Selection of an optimal therapy according to proper risk stratification in NKTCL is necessary to further improve the clinical outcome.

Several prognostic models for NKTCL including the International Prognostic Index (IPI) and Korean Prognostic Index (KPI), and positron emission tomography/ computed tomography (PET/CT) related parameters such as maximum standard uptake (SUVmax) have been proposed to accurately predict the clinical outcome [9, 10]. Furthermore, recent study reported that combined analysis of both circulating EBV DNA and post-treatment PET-CT response could predict recurrence of NKTCL in patients treated with current chemoradiotherapy or non-anthracycline-based chemotherapy [11]. However, their predictive values for the survival remain controversial. Other prognostic models studied for NKTCL include the Prognostic Index for T-cell lymphoma (PIT). This new model combined the KPI, Glasgow Prognostic Score (GPS) and metabolic tumor volume (MTV) on PET/CT [12-14].

Tumor necrosis (TN) is a recognized consequence of chronic hypoxic injury of cancer cells due to rapid tumor growth. Increased cellular hypoxia in cancer is associated with increased potential of distant metastasis and resistance to RTx and CTx [15, 16]. Because NKTCL is an angiocentric angio-destructive lymphoproliferative neoplasm, TN is a common clinical feature in NKTCL. However, the clinical importance of TN on survival is unknown.

Examination of TN as a meaningful negative prognostic factor interfering with RTx or CTx in NKTCL is necessary. This retrospective study investigated the clinical impact of TN on survival in patients with nasal NKTCL, as well as the importance of other clinical prognostic parameters.

## RESULTS

### Patient characteristics

The clinical characteristics of the 100 patients with limited stage nasal NKTCL are summarized in Table 1. Seventy-seven patients (77.0 %) were male and 45 patients (45.0 %) were > 60 years of age. Thirty-one patients (31.0 %) have regional nodal involvement and poor Eastern Cooperative Oncology Group (ECOG) performance status (PS) above grade 2 was present in 12 patients (12.0 %). Elevated lactate dehydrogenase (LDH) level was present in 29 patients (29.0 %) and B symptoms was present in 21 patients (21.0 %). Local tumor invasiveness was present in 22 patients (22.0 %).

**Table 1:** Baseline characteristics in patients with ENKTL

Characteristics	Total (*n* = 100)	TN group (*n* = 41)	No TN group (*n* = 59)	*p*-value
**Gender**				
Male	77 (77.0)	29 (29.0)	48 (48.0)	0.541
Female	23 (23.0)	12 (12.0)	11 (11.0)	
**Age (years)**				
> 60 years	45 (45.0)	20 (20.0)	25 (25.0)	0.529
≤ 60 years	55 (55.0)	21 (21.0)	34 (34.0)	
**Regional nodal involvement**				
Yes	31 (31.0)	16 (16.0)	15 (15.0)	0.150
No	69 (69.0)	25 (25.0)	44 (44.0)	
**ECOG PS**				
≥ 2	12 (12.0)	8 (8.0)	4 (4.0)	0.049
0-1	88 (88.0)	33 (33.0)	55 (55.0)	
**Serum LDH level**				
High than normal	29 (29.0)	15 (15.0)	14 (14.0)	0.166
Normal	71 (71.0)	26 (26.0)	45 (45.0)	
**B symptoms**				
Yes	21 (21.0)	12 (12.0)	9 (9.0)	0.092
No	79 (79.0)	29 (29.0)	50 (50.0)	
**IPI score**				
0-2	91 (91.0)	35 (35.0)	56 (56.0)	0.102
≥ 3	9 (9.0)	6 (6.0)	3 (3.0)	
**Local tumor invasiveness**				
Yes	22 (22.0)	10 (10.0)	12 (12.0)	0.630
No	78 (78.0)	31 (31.0)	47 (47.0)	
**Korean prognostic Index (KPI)**				
High KPI score ≥ 3 points	11 (11.0)	8 (8.0)	3 (3.0)	0.024
**Glasgow prognostic score (GPS)**				
High GPS (2 points)	31 (31.0)	17 (17.0)	14 (14.0)	0.061
**CRP/albumin ratio (CAR)**				
High CAR	22 (22.0)	15 (15.0)	7 (7.0)	0.003
**SUVmax on PET/CT**				
Median (range)	6.4 (2.9-22.3)	8.4 (2.9-21.1)	5.2 (3.1-22.3)	0.008
**Metabolic tumor volume on PET/CT**				
Median (range)	36.2 (5.1-1164.9)	84.5 (5.1-1164.9)	32.8 (6.2-337.0)	<0.001
**Treatment**				
**Complete resection group**	37 (37.0)	13 (13.0)	24 (24.0)	0.363
CMT	8 (8.0)	3 (3.0)	5 (5.0)	0.399
Chemotherapy only	29 (29.0)	10 (10.0)	19 (19.0)	
**Only biopsy group**	63 (63.0)	28 (28.0)	35 (25.0)	0.363
CMT	56 (56.0)	26 (26.0)	30 (30.0)	0.490
Chemotherapy only	7 (7.0)	2 (2.0)	5 (5.0)	

ECOG, Eastern Cooperative Oncology Group; PS, performance status; LDH, lactate dehydrogenase; IPI, International Prognostic Index; SUVmax, maximum standard uptake value; PET/CT, positron emission tomography; CT, computed tomography, CMT, combined modality treatment (concurrent chemoradiotherapy with chemotherapy)

We also analyzed distribution of the patients based on famous prognostic scores such as KPI, Glasgow prognostic score (GPS) and serum C-reactive protein/albumin ratio (CAR). Eleven patients (11.0 %) have high KPI score (≥ 3 points) and 31 patients (31.0 %) have high GPS (2 points). Determination for cut-off value between High and low CAR were performed by Receiver operating characteristic (ROC) curve analysis. According to ideal cut off value, 0.21 by the analysis, 22 patients (22.0 %) have high CAR (CAR ≥ 0.21) (Table 1).

### Clinical characteristics and clinical impact of TN and complete resection

**Figure 1:**
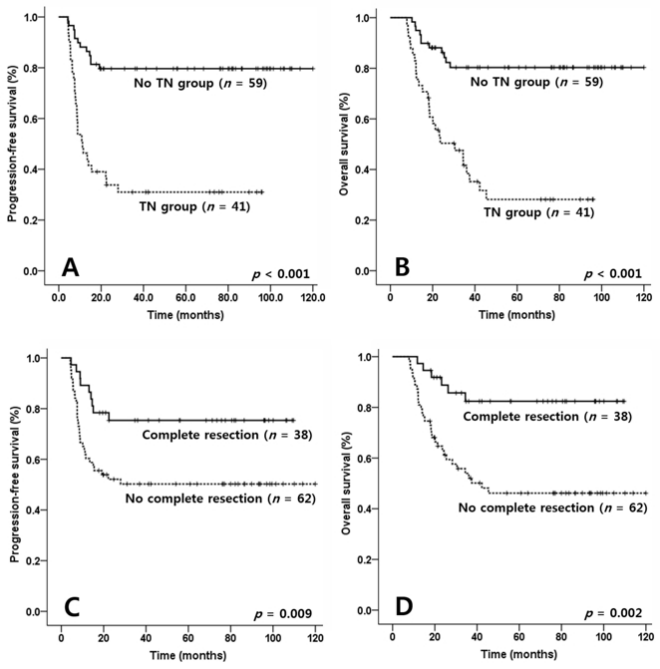
Comparisons of progression-free survival (PFS) and overall survival (OS) according to tumor necrosis (TN) and complete resection in patients with NK/T cell lymphoma (NKTCL) PFS and OS of TN group were inferior compared with no TN group in patients with NKTCL (*p* < 0.001, *p* < 0.001, Figure 1A & 1B). Meanwhile, survivals of complete resection group were superior compared with no complete resection group in the patients (*p* = 0.009, *p* = 0.002, Figure 1C & 1D).

Forty-one patients displayed TN group and 59 patients did not. Adverse features that were significantly more prevalent in those with TN included poor ECOG PS above than grade 2 (*p* = 0.049), high KPI score (*p* = 0.024), high CAR (*p* = 0.003), higher SUVmax (*p* = 0.008) and higher MTV on PET/CT (*p* < 0.001) (Table 1). Survival was compared in the TN and no TN groups to ascertain the involvement of TN with worse survivals in addition to the aforementioned adverse features. Kaplan-Meier curves revealed shorter progression-free survival (PFS) and overall survival (OS) in the TN group (*p* < 0.001 and *p* < 0.001, respectively; Figures 1A and 1B).

Fifty-six (56.0 %) in only biopsy group and only 8 (8.0 %) in complete resection group received concurrent chemoradiotherapy. The complete resection group had relatively lower radiation exposure rate than only biopsy group (median 20.0 Gy in complete resection group versus median 45.0 Gy in only biopsy group, *p* < 0.001, data not shown). However, PFS and OS in complete resection group were superior compared with only biopsy group (*p* = 0.009 and *p* = 0.002, respectively; Figures 1C and 1D).

### Clinical values of prognostic factors on survival

Clinical values of age > 60 years, male sex, regional LN involvement, poor ECOG PS ≥ grade 2, elevated LDH level, B symptoms, local tumor invasiveness, high KPI score ≥ 3 points, high GPS (2 points), high CAR (≥ 0.21), high SUVmax, high MTV status, TN and complete resection on survivals were analyzed. To determine ideal cut-off values of the SUVmax and MTV on survivals, the analysis ROC curve analysis was performed. The analysis indicated that ideal cut-off value of SUVmax and MTV was 8.5 and 94.2 cm^3^, respectively (sensitivity versus [vs.] specificity, 90.3% vs. 64.4% in SUVmax; 90.1% vs. 70.3% in MTV; data not shown).

To analyzed the clinical impact of the above prognostic factors on survivals, univariate analysis was performed. In the analysis, elevated LDH level (*p* = 0.034; *p* = 0.045), poor ECOG PS ≥ grade 2 (*p* = 0.047; *p* = 0.021), high SUV max ≥ 8.5 (*p* = 0.002; *p* = 0.007), high MTV ≥ 94.2 cm3 (*p* < 0.001; *p* < 0.001), TN (*p* < 0.001; *p* < 0.001), local tumor invasiveness (*p* < 0.001; *p* < 0.001), complete resection (*p* = 0.009; *p* = 0.002), regional LN involvement (*p* < 0.001; *p* < 0.001) and presence of B symptoms (*p* = 0.017; *p* = 0.018), high KPI score (*p* = 0.012; *p* = 0.033), high CAR (*p* < 0.001; *p* < 0.001) and high GPS (*p* = 0.009; *p* = 0.007) were significantly associated with PFS and OS in limited stage nasal NKTCL (Table 2).

**Table 2:** Univariate and multivariate analysis of prognostic factors in patients with ENKTL

	Progression-free survival			Overall survival		
	Univariate	Multivariate		Univariate	Multivariate	
	*P*-value	HR (95% CI)	*p*-value	*P*-value	HR (95% CI)	*p*-value
**Age > 60 years**	0.515	—	—	0.605	—	—
**Male sex**	0.607	—	—	0.504	—	—
**Elevated LDH level**	0.034	2.093 (0.780-5.614)	0.142	0.045	0.891 (0.354-2.239)	0.805
**ECOG PS above grade 2**	0.047	0.804 (0.274-2.354)	0.690	0.021	0.693 (0.208-2.307)	0.550
**High SUVmax on PET/CT**	0.002	1.796 (0.881-3.663)	0.107	0.007	0.614 (0.257-1.468)	0.273
**High MTV status**	<0.001	3.810 (1.751-8.293)	0.001	<0.001	2.473 (1.492-6.162)	0.032
**Tumor necrosis**	<0.001	3.015 (1.210-7.508)	0.018	<0.001	3.482 (1.365-8.888)	0.009
**Local tumor invasiveness**	<0.001	2.962 (1.347-6.513)	0.007	<0.001	2.484 (1.067-5.787)	0.035
**Complete resection**	0.009	0.299 (0.108-0.829)	0.020	0.002	0.305 (0.106-0.879)	0.028
**Regional LN involvement**	<0.001	5.728 (2.428-13.513)	<0.001	<0.001	5.037 (2.284-11.203)	<0.001
**B symptoms**	0.017	0.408 (0.148-1.122)	0.082	0.018	1.301 (0.417-4.057)	0.650
**High KPI score**	0.012	0.685 (0.187-2.510)	0.568	0.033	0.407 (0.102-1.620)	0.202
**High CAR**	<0.001	2.038 (0.849-4.881)	0.111	<0.001	1.461 (0.591-3.611)	0.411
**High GPS**	0.009	0.853 (0.393-1.848)	0.686	0.007	1.758 (0.792-3.902)	0.165

LDH, lactate dehydrogenase; ECOG, Eastern Cooperattive Group; PS, performance status; EN, extranodal; SUVmax, maximum standard uptake value; PET/CT, positron emission tomography/computed tomography; MTV, metabolic tumor volume; LN, lymph node; KPI, Korean Prognostic Index; CAR, C-reactive protein/albumin ratio; GPS, Glasgow prognostic score.

In order to measure independent clinical impacts of significant prognostic factors in univariate analysis on survivals, multivariate analysis was performed. High MTV status (PFS, HR = 3.810, 95% CI = 1.751 – 8.2935, *p* = 0.001; OS, HR = 2.473, 95% CI = 1.492 – 6.162, *p* = 0.032), TN (PFS, HR = 3.015, 95% CI = 1.210 – 7.508, *p* = 0.018; OS, HR = 3.482, 95% CI = 1.365 – 8.888, *p* = 0.009), local tumor invasiveness (PFS, HR = 2.962, 95% CI = 1.347 – 6.513, *p* = 0.007; OS, HR = 2.484, 95% CI = 1.067 – 5.787, *p* = 0.035), complete resection (PFS, HR = 0.299, 95% CI = 0.108 – 0.829, *p* = 0.020; OS, HR = 0.305, 95% CI = 0.106 – 0.879, *p* = 0.028) and regional LN involvement (PFS, HR = 5.728, 95% CI = 2.428 – 13.513, *p* < 0.001; OS, HR = 5.037, 95% CI = 2.284 – 11.203, *p* < 0.001) were independently associated with survival in patients with limited stage nasal NKTCL.

### High volume TN and unresected TN predicts worse survival

**Figure 2:**
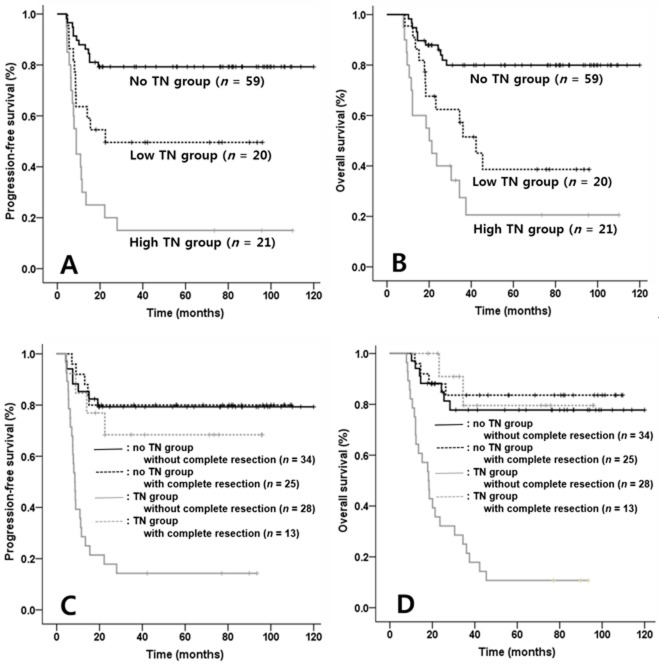
Comparisons of progression-free survival (PFS) and overall survival (OS) according to extent of tumor necrosis (TN) and combined two factors such as TN and complete resection in patients with NK/T cell lymphoma (NKTCL) Each PFS and OS among three groups divided according to extent of TN was significantly different in patients with NKTCL (PFS & OS between No TN & low NTV group, *p* = 0.027 & *p* = 0.007; PFS & OS between low NTV and high NTV group, *p* = 0.004 & *p* = 0.025, Figure 2A & 2B). Meanwhile, in comparisons according to combined two factors including TN and complete resection, TN group not received complete resection had lowest PFS and OS than other three groups (*p* < 0.001, *p* < 0.001, Figure 2C & 2D).

In order to identify the extent of TN that adversely affect survival, depending on median value of NTV as the cut-off value (median value, 97.1 cm^3^; range, 2.3 – 789.3 cm^3^; data not shown), the TN group was divided into high NTV (≥ 97.1 cm^3^, *n* = 21), low NTV (<97.2 cm^3^, *n* = 20) and no TN group (*n* = 59). PFS and OS among the three groups were compared and Kaplan-Meier survival curves were showed that PFSs were significantly different between no TN and low NTV group (*p* = 0.036) and between low and high NTV group (*p* = 0.002; Figure 2A). Similarly, OSs were different between no TN and low NTV group (*p* = 0.008) and between low and high NTV group (*p* = 0.010; Figure 2B).

We also compared the combined clinical impacts of TN and complete resection as independent prognostic factors on survival. In the survival analysis, TN group without complete resection displayed inferior PFS and OS compared with other three groups (PFS, *p* < 0.001, Figure 2C; OS, *p* < 0.001, Figure 2D). Differences of survivals among other three groups (TN group with complete resection, no TN group with complete resection and no TN group without complete resection) were not present.

## DISCUSSION

High MTV status, TN, local tumor invasiveness, complete resection and regional LN involvement were independently associated with PFS and OS in patients with nasal NKTCL. Local tumor invasiveness and regional LN involvement are clinical prognostic factors [12, 17]. Their clinical impact on survival was reaffirmed here. High MTV status as a measure of high tumor burden in NKTCL also had reliable prognostic values presently and in a recent clinical study [14].

The results implicate TN and complete resection as novel meaningful prognostic factors could significantly predict survival in NKTCL. TN is mainly caused by tissue hypoxia due to rapid tumor growth, insufficient neovascularization, compression and thrombotic obstruction of adjacent vessels of cancer. Recently, histopathologically-identified TN has been considered as an important prognostic factor for several solid cancer types including breast, lung, pancreas and kidney [15, 18-20]. We also found significant differences in survival between TN and no TN group in patients with (PFS, 79.7 % & OS, 81.4 % in no TN group versus 31.7 % and 34.1 % in TN group, respectively). Thus, TN could influence survival in UAT NKTCL.

FDG uptake of necrotic area is generally low due to cancer cell death. However, the present study showed that SUVmax in TN group was higher than non-TN group. The value of SUVmax at peri-necrotic area in many cases was high, even if the central necrotic area was low. TN is well-known to be significantly associated with increased angiogenesis, inflammation and cell proliferation which reflect tumor aggressiveness [21]. Therefore, we hypothesized that the excessive inflammation and angiogenesis stimulated by the tissue hypoxia could actively occur in peri-necrotic area, and ultimately increase metabolic activity of the area. Similarly, a previous laboratory study [22] demonstrated that newly formed granulation tissue and tumor-associated macrophages infiltrating the marginal areas surrounding the necrotic area of the tumor show a high uptake of 18F-FDG.

The extent of TN also affected prognosis in patients with NKTCL. Survival in the no TN group and the low and high necrotic tumor volume (NTV) subgroups based on median value of NTV were significantly different. Therefore, a higher extent of TN might be correlated with inferior survival in NKTCL. High NTV based on CT scan was reported to be lower in patients with recurrent head and neck cancer than control patients [23]. Tumors with less extensive necrosis (< 4 cm^3^) were associated with good prognosis regardless of the type of treatment. In several cancer states other than NKTCL, some studies have similarly described that larger TN volume reflects aggressive behavior and poor survival in patients [24, 25].

Although NKTCL is a radiosensitive lymphoma subtype, the optimal treatment strategy for nasal NKTCL is not clear. Hypoxic volume has been reported to be the strongest predictor of the response to RT and survival [26, 27]. TN could have a negative impact on survival by reducing radio-sensitivity. Necrotic components due to the discriminative angiocentric and angio-destructive propensity of NKTCL could diminish the efficacy of treatment. Therefore, a novel treatment strategy able to overcome the drawback of current treatment strategies is needed. Presently, the survival in complete resected patients with NKTCL was meaningfully superior compared with other patients not receiving complete resection, especially compared to the no TN group. In a prior study, only 3 of the cohort of 26 NKTCL patients received surgical resection [28]. Interestingly, these 3 patients were the only long-term survivors. Although clinical data about the prognostic value of surgical resection in NKTCL are limited, surgical resection in NKTCL may be linked with a favorable prognosis.

Concurrent chemoradiotherapy is one of optimal therapy in early stage NKTCL and has showed an excellent response rate and favorable survival benefits [7, 8]. However, malnutrition due to significant painful mucositis and xerostomia during or after concurrent chemoradiotherapy leads to adverse consequences including poor quality of life, decreased immune function and shorter survivals [29-31]. Nutritional deficiency in elderly patients is more frequent than young patients and previous data showed that older patients received little benefit from concurrent chemoradiotherapy and had lower tolerance and increased toxicity of the treatment [32-34]. Because median age at the time of diagnosis of UAT NKTCL was reported to be from 50 to 60 years, many of those would be included in elderly patients above than 60 years [35]. Therefore, the above adverse features from concurrent chemoradiotherapy might reduce response rate and survivals in UAT NKTCL, although it was reported be tolerable treatment modality.

Endoscopic resection and robotic surgery have shown excellent treatment results, quick recovery and very low sequelae in head and neck cancer [36, 37]. Therefore, surgical resection in nasal NKTCL could be an alternate treatment modality in patients with extensive TN not eligible for RTx or CTx.

Evaluation of the combined prognostic values of TN and complete resection as independent factors on survival showed that patients with TN without complete resection had inferior survival. Moreover, based on Figure 2C and 2D, the results showed that clinical impact of TN was maximized in patients who did not receive complete resections compared with those received complete resection. Therefore, the existence of unresected TN appears to have important prognostic significance on the survival in patients with nasal NKTCL. However, the present study has a limitation that TN is affirmed by only imaging modality, but not pathologic confirmation. Therefore, we regard that the pathologic identification for TN would be needed to confirm the clinical impact in NKTCL.

In conclusion, TN and complete resection are novel prognostic factors with clinical impact on survival in nasal NKTCL. Especially, TN contained lymphoma mass in the nasal cavity could counteract the clinical effect of RTx and CTx. TN should be eliminated completely to improve survival in nasal NKTCL. Further well designed clinical studies are warranted to confirm our results.

## PATIENTS AND METHODS

### Patient eligibility

From April 2006 to July 2015, 100 patients with limited stage nasal NKTCL were enrolled from four institutes. The patients were newly diagnosed as limited stage nasal NKTCL and had not been previously treated for the disease. The patients received concurrent chemoradiotherapy with or without non-anthracycline-based CTx or non-anthracycline-based CTx only. Patients with non-nasal NKTCL, those with other malignant diseases and those who received anthracycline-based CTx were excluded. Moreover, those who received autologous stem cell transplantation as a consolidation treatment were excluded in the present study.

All patients underwent staging procedures including physical examination, complete blood cell counts and blood chemistry, bilateral bone marrow biopsy, and imaging study including CT scan, magnetic resonance imaging and ^18^F-FDG-PET/CT scan. Nasal involvement was affirmed by the imaging studies. All pathologic specimens were classified based on strict morphologic criteria along with immunophenotypic findings (World Health Organization classification). Patient data were obtained by review of electric medical records by local doctors. Approval for the retrospective review of these records was obtained from the Institutional Review Boards of all participating medical centers.

### Assessment of MTV on PET/CT

Dual-modality PET/CT tomography was performed using a biograph (Siemens Medical Solution, Hoffman Estates, IL, USA) according to the major guideline for standard oncological PET imaging [38]. Briefly, the patients fasted for at least 6 h prior to the intravenous administration of ^18^F-FDG (7.4 MBq per body weight) to ensure a serum glucose level below 7.2 mmol/L. At 60 min after ^18^F-FDG administration, transmission data were acquired using low-dose CT (120 kV, automated from 10 to 130 mA, a 512 × 512 matrix, a 50-cm field of view (FOV), 3.75-mm slice thickness, and a rotation time of 0.8 s), extending from the base of the skull to the proximal thighs. Immediately after CT acquisition, PET emission scans were acquired in the same anatomic locations with a 15.7-cm axial FOV acquired in the two-dimensional mode with a 128 × 128 matrix. The CT data were used for attenuation correction. The images were reconstructed using a conventional iterative algorithm (OSEM). A workstation (AW Volume Share™) providing multi-planar reformatted images was also used for image display and analysis. A target area having SUV ≥ 2.5 was defined as a lymphoma involvement area [39, 40]. Among all lesions detected on the PET/CT scan, highest FDG uptake valued area was defined as SUVmax. MTV regions on PET images were evaluated for nodal and EN area having the increased tracer uptake in patients with NKTCL. CT images of PET/CT were used for the attenuation correction. Corrected emission data images were reconstructed after Fourier transformation with AWOSEM software (2 iterations, 8 subsets, 5 mm Gaussian filter)

### Definition and volumetric assessment of tumor necrosis in UAT NKTCL

The TN area on CT scans was defined as an area of diminished or non-enhancement of the mass lesion after intravenous administration of contrast material. TN in the lymph node was not considered in this measurement. The TN group comprised patients with TN detected on CT scan or MRI at the time of diagnosis. The volume of necrotic tumor area (NTV) was measured with manual segmentation (Voxar 3D ActiveX; Toshiba Medical Visualization Systems, Edinburgh, Scotland) [41]. All CT data were independently reviewed by two radiologists. The assessment of TN was decided by consensus agreement. Subsequently, the imaging data of patients with TN was centrally reviewed by expert radiologists.

### Treatment modality

The treatment option was selected according to physician judgment at each medical center. The only biopsy group comprised 63 patients confirmed only by tissue biopsy at a pathologic lesion, such as a nasal mass or pathologic LN of NKTCL. The complete resection group included 37 patients who were confirmed by complete resection for the primary EN lymphoma mass within UAT, but not LN.

Fifty-six of the 63 only biopsy group received concurrent chemoradiotherapy followed by non-anthracycline-based CTx. Concurrent chemoradiotherapy was performed by weekly cisplatin administration. (RTx, median, 45.0 Gy; range, 38.0-50.0 Gy). The remaining 7 received only non-anthracycline based CTx. (median 6 cycles were performed in only biopsy group; range 5 - 8 cycles). Eight complete resection group patients received concurrent chemoradiotherapy for remnant lesions after discontented resection. (median, 20.0 Gy; range, 18.0-23.0 Gy). The remaining 29 complete resection patients received only non-anthracycline-based CTx (median 6 cycles were performed in complete resection group; range, 5 - 8 cycles). Non-anthracycline CTx regimens were ifosfamide, methotrexate, and etoposide (IMEP, 19 patients received); ifosfamide, etoposide, dexamethasone, and L-asparaginase (VIDL, 56 patients received); ifosfamide, etoposide, cisplatin and dexamethasone (VIPD, 25 patients received). The above 29 complete resection patients were treated with the CTx such as IMEP (6 patients), VIPD (6 patients), and VIDL (17 patients).

### Statistical analyses

The Mann–Whitney U-test was used to compare differences in manifestations of prognostic factors in patients with nasal NKTCL. ROC curve estimated the accuracy in predicting the ideal cut-off value of CAR, SUVmax and MTV. Estimation of sensitivity and specificity was based on the cut-off value of MTV. PFS was calculated from the date of diagnosis to document disease progression. OS was calculated from the date of diagnosis until either death as a result of any cause, or the date last known to be alive. Kaplan-Meier method was used to evaluate PFS and OS. By log-rank test, differences of the survivals were compared. Statistical analysis was carried out with SPSS software version 18.0 (SPSS Inc., Chicago, IL). A probability value <0.05 was considered statistically significant.
